# Exploring the influence of medical staffing and birth volume on observed-to-expected cesarean deliveries: a panel data analysis of integrated obstetric and gynecological departments in Germany

**DOI:** 10.1007/s10198-024-01749-0

**Published:** 2025-01-21

**Authors:** Arno Stöcker, Holger Pfaff, Nadine Scholten, Ludwig Kuntz

**Affiliations:** 1 Faculty of Human Sciences and Faculty of Medicine and University Hospital Cologne, Institute of Medical Sociology, Health Services Research and Rehabilitation Science, Chair of Quality Development and Evaluation in Rehabilitation, University of Cologne, Cologne, Germany; 2 Faculty of Medicine and University Hospital Cologne, Institute of Medical Sociology, Health Services Research and Rehabilitation Science, Chair of Health Services Research, University of Cologne, Cologne, Germany; 3 Department of Business Administration and Health Care Management, Faculty of Management, Economics and Social Sciences, University of Cologne, Cologne, Germany; 4 Center for Health Services Research Cologne, Interfaculty Institution of the University of Cologne, Cologne, Germany; 5 Center for Health Communication and Health Services Research, Department for Psychosomatic Medicine and Psychotherapy, Faculty of Medicine, University Hospital Bonn, Bonn, Germany

**Keywords:** Longitudinal design, Obstetric care, Inpatient sector, Hospital, Organizational management, Medical staffing, C23, I10, N34, P46

## Abstract

**Introduction:**

Cesarean deliveries account for approximately one-third of all births in Germany, prompting ongoing discussions on cesarean section rates and their connection to medical staffing and birth volume. In Germany, the majority of departments integrate obstetric and gynecological care within a single department.

**Methods:**

The analysis utilized quality reports from German hospitals spanning 2015 to 2019. The outcome variable was the annual risk-adjusted cesarean section ratio—a metric comparing expected to observed cesarean sections. Explanatory variables included annual counts of physicians, midwives, and births. To account for case number-related staffing variations, full-time equivalent midwife and physician staff positions were normalized by the number of deliveries. Uni- and multivariate panel models were applied, complemented by multiple instrument variable analyses, including two-stage least square and generalized method of moments models.

**Results:**

Incorporating data from 509 integrated obstetric departments and 2089 observations, representing 2,335,839 deliveries with 720,795 cesarean sections (over 60% of all inpatient births in Germany), multivariate model with fixed effects revealed a statistically significant positive association between the number of physicians per birth and the risk-adjusted cesarean section ratio (0.004, p = 0.004). Two-stage least square instrument variable analysis (0.020, p < 0.001) and a system GMM estimator models (0.004, p < 0.001) validated these results, providing compelling evidence for a causal relationship.

**Conclusion:**

The study established a robust connection between the number of physicians per birth and the risk-adjusted cesarean section ratio in integrated obstetric and gynecological departments in Germany. While the cause of the effect remains unclear, one possible explanation is a lack of specialization within these departments due to the combined provision of both obstetric and gynecological care.

## Introduction

Over the past few decades, the global prevalence of cesarean section (C-section) births has steadily increased [1]. Some experts and researchers go so far as to characterize this trend as an endemic or pandemic of C-sections [2, 3]. This is noteworthy given that vaginal delivery is generally regarded as the preferred method compared to cesarean birth [4]. In Germany, the C-section rate has doubled since 1991 and has stabilized at approximately 30% since the mid-2000s [5]. Thereby, indications for a cesarean section can be divided into absolute and relative indications. This increase is predominantly attributed to relative indications, such as breech presentation, birth arrest, impending fetal hypoxia, and post-section conditions [6], with 90% of C-sections lacking absolute medical indications [7]. Thus, understanding non-medical factors, including organizational influences, is imperative for a comprehensive study of delivery methods.

Given the imperative for health care providers to align their actions with medical necessities and patient preferences, comprehending the factors influencing medical practice decisions is crucial. In the case of C-sections, obviously maternal desire is a key factor [6, 8–10]. From the provider's perspective, numerous factors influence the choice of birth method. These encompass individual factors of the physician [3, 8, 11–14] or midwife [15, 16], as well as organizational factors at the hospital or obstetric department level [13, 17–22]. Additionally, context-related factors, such as the complexities of caseload [23], contribute to the decision-making process. Studies on C-section rates and the quality of obstetric care reveal organization-specific differences linked to factors such as hospital ownership, number of beds, and teaching activity, both in Germany [17–19, 24, 25] and internationally [26–30].

While variations between departments with different organizational factors have been extensively studied, understanding relationships and interactions within obstetric departments is equally vital. These inter-organizational influences can potentially impact all departments uniformly, independent of specific organizational factors such as culture, region, socio-demographics, and patient population effects. From an organizational standpoint, comprehending these influences is crucial for making informed decisions about medical practices. Organizations can enhance their understanding of the factors shaping medical practice within their purview and implement adjustments accordingly [31].

This analysis focuses on two organizational factors, namely, medical staffing and caseload volume, and explores their interplay. Both factors have been acknowledged for their influence on the quality and quantity of obstetric care in general [27, 32] and specifically on C-sections (e.g., volume of births [33, 34] or the number of physicians/deliveries per physician per year [35–37]). Overall, medical staffing levels have been less frequently investigated than birth volume as an explanatory variable. Our study enhances current understanding through a longitudinal investigation that encompasses a significant portion of German obstetric departments and deliveries. Importantly, by employing an externally evaluated risk-adjusted C-section ratio, our analysis addresses methodological limitations by accounting for medical indications on the part of both mother and child. Consequently, the ratio and the analysis remain robust, mitigating biases induced by underlying medical reasons that may prompt a C-section [38].

Our research question delved into how the number of full-time equivalent physicians and midwives per birth, coupled with the volume of births, impact the risk-adjusted C-section ratio in German combined obstetric and gynecological departments. In doing so, we address methodological shortcomings by focusing on differences within hospital departments, contributing to the understanding of factors independent of variances between hospital departments. Utilizing a panel model data set with individual and time effects, we control for department- and patient population-specific characteristics, ensuring a comprehensive analysis [39].

## Methods

### Data source

This analysis was based on the structured quality reports of German hospitals, which serve as a cornerstone of transparency in the inpatient sector in Germany [40–44]. These reports are compiled and disclosed in accordance with Sects. 136 and 137 of the German Social Security Code (Sozialgesetzbuch) V. They offer a comprehensive overview of the structures, services, and quality of the respective hospital and its specialist departments. Annual reports include documentation of performance, organizational metrics, and various quality indicators and scores. Details are delineated to the department level. Although this data set has been utilized for cross-sectional analyses of various indicators [18, 40–42, 45, 46], its application in the context of longitudinal analyses with a specific focus on obstetrics is novel. During the study period, approximately 30 quality indicators related to obstetric care were published (Appendix Table 7). Our analysis centers on the quality indicator for risk-adjusted C-section rates per hospital.

In preparation for this analysis, a panel data set was compiled using annually published quality reports. In these reports each hospital is assigned a unique identification code, and each site within a hospital is designated a site number. As previously outlined [47, 48], identification codes and site numbers may undergo changes over the years. Therefore, automated linking without content verification could yield a success rate ranging from 80 to 90%. Recognizing potential changes in identification codes and site numbers over the years, a manual matching process was employed to ensure accuracy, supplementing a machine linkage via identification codes and site numbers. The linkage incorporated hospital and site addresses, bed numbers, and the names of responsible department and general hospital managers. Two independent researchers performed the manual linkage, resolving disputed assignments through consensus. Each hospital and site received a master identification number, forming the foundation for the panel study.

As this analysis employs secondary data, and the data are publicly available, no ethical committee vote was deemed necessary.

### Study population

In Germany, the vast majority of deliveries occur in hospitals, with less than two percent taking place at home or in birthing centers. Obstetric care is predominantly administered in departments specializing in both obstetrics and gynecology simultaneously. Consequently, two closely related but increasingly distinct medical services are offered within the same departments. Given the inherent organizational disparities between those integrated obstetrics departments and departments primarily dedicated on obstetric care, we excluded obstetric departments with a primary focus on obstetric care from our analysis. Importantly, there are no systematic differences between these two types of departments in terms of patient population or medical standards. The cut-off value was determined through a data-driven approach, as information on organizational focus was not consistently or continuously included in the quality reports. Additionally, departments with attending physicians were excluded due to their typically higher C-section rates in Germany [17, 18, 25].

Ensuring data integrity, we identified potential outliers through Cook's distance and subsequently verified them manually. Closed departments were confirmed through cross-referencing with press reports. The exclusion was deemed necessary to avoid inaccuracies resulting from scenarios where a department, operational for only six months, reported staffing numbers analogously for the entire year. Consequently, staffing figures were not proportionately reported in the quality report, and the number of deliveries was only accounted for during the specified 6-month period. This meticulous approach was adopted to maintain the accuracy and reliability of the dataset.

### Study period

The observation period spans from 2015 to 2019, as the relevant quality indicator on the cesarean section ratio was introduced in 2015, and 2019 marks the last year before the onset of the COVID-19 pandemic. Recognizing the potential impact of measures associated with the pandemic on daily hospital practices [49, 50], data from 2020 onwards was excluded to minimize bias. Moreover, the introduction of the Robson indicator to the quality indicator in 2020 [51] further complicates longitudinal comparisons.

## Measures

### Outcome variable

The dependent variable in the study was the risk-adjusted cesarean section ratio at the department level (quality indicator no. 52249). This ratio is calculated as the observed number of C-sections divided by the expected number of C-sections. The declared quality objective is the minimization of cesarean births [51]. The numerator includes all observed cesarean deliveries within a department, while the denominator comprises risk-adjusted expected cesarean deliveries. In 2015 and 2016, all mothers with at least one child born after 24 weeks were included, while from 2017 onwards, only mothers delivering between weeks 24 and 42 were considered. Risk adjustment is performed by the Institute for Quality Assurance and Transparency in Healthcare (Institut für Qualitätssicherung und Transparenz im Gesundheitswesen [IQTIG]). While individual patient data are considered in this process, only aggregated, annual department-level data were accessible for this analysis open access.

Notably, the researchers involved in this study were not directly engaged in the risk adjustment process. The selection of risk factors was guided by Becker and Eissler [52] in collaboration with the Federal Perinatal Medicine Group. Risk factors considered encompassed variables such as maternal age, comprehensive data on infant and maternal health status, and information on previous deliveries (Appendix Table 22). Each year, these risk factors were adjusted based on their respective regression coefficients. Consequently, the risk adjustment undertaken compensates for the divergent patient structures across different facilities, offering a more equitable basis for facility comparisons. This adjustment proves pivotal in ensuring a fair assessment, as patients bring individual risk factors, including concomitant diseases, that could systematically influence the quality outcome. Through risk adjustment, institutions with a higher prevalence of high-risk cases can be statistically juxtaposed more equitably with those handling a larger proportion of low-risk cases, thereby facilitating an unbiased analysis [53]. In the context of the risk-adjusted C-section ratio, values below 1 indicate that a department is performing fewer cesarean sections than expected. Conversely, a ratio above 1 signifies that more risk-adjusted cesarean sections are being performed than anticipated. For instance, a C-section ratio of 1.1 would imply that 10% more cesarean sections were performed than expected. Furthermore, a reference category is defined for each year, designating departments above the 90th percentile as conspicuous. The corresponding value for the review period ranged from 1.23 to 1.27.

### Explanatory variables

Three independent variables were utilized: (1) the number of deliveries per department (mothers giving birth) per 1000, (2) the number of full-time equivalent physicians in the department per 1000 deliveries, (3) and the number of full-time equivalent midwives in the department per 1000 deliveries. Staffing levels have been divided by 1000 to account for volume related differences in the departments. The data on deliveries were extracted from the denominator of the quality indicator for each department. The data on both staffing levels were presented separately for each department within a hospital. Staffing data for physicians were aggregated for residents and specialists, collectively referred to as physicians in subsequent discussions. Physician assistants were excluded. As these departments integrate obstetrics and gynecology, physicians represent both specialties. In Germany, the ‘Facharztstandard’ (specialist standard) ensures high-quality medical treatments and procedures, typically carried out or supervised by a specialist. However, sufficiently trained residents or assistant physicians may also perform treatments and procedures independently, including cesarean sections. Since there is no strict threshold for determining when a physician has gained sufficient experience and knowledge, we opted to include the total number of physicians in our variable. In addition, a midwife has to be present during labor in Germany (‘Hinzuziehungspflicht’; midwife's obligation to consult).

### Control variables

Three organizational variables served as control variables in the analyses: (1) hospital ownership (non-profit, private, public), (2) teaching status (no teaching assignment, academic teaching hospital, university hospital), and (3) perinatal care level (regular obstetric departments [care level 4], perinatal focus [care level 3], perinatal center level II [care level 2], perinatal center level I [care level 1]). Regular obstetric departments provide standard perinatal care from 36 + 0 weeks of gestation without anticipated complications. Departments with a perinatal focus cater to pregnant women expecting premature infants with an estimated birth weight of at least 1500 g or with a gestational age from 32 + 0 to less than or equal to 35 + 6 weeks. Level II perinatal centers serve pregnant women with anticipated premature infants weighing between 1250 and 1499 g or with a gestational age from 32 + 0 to less than or equal to 35 + 6 weeks. Level I perinatal centers offer the highest level of obstetric care for pregnant women expecting premature infants with an estimated birth weight under 1250 g or with a gestational age from 29 + 0 to less than weeks. All three organizational variables were frequently cited as influential factors in C-section rates [54, 55].

### Model description

We developed various static and dynamic panel models featuring time- and individual-specific effects (two-way effects), employing cluster-robust estimators for each department:

- Fixed effects estimator models
  $$y_{it} = \beta_{0} + \beta_{1} x_{it} + \gamma_{t} + \alpha_{i} + e_{it}$$
- Correlated random effects estimator models
  $$y_{it} = \beta_{0} + \beta_{1} x_{it} + \beta_{2} \overline{x}_{i} + \beta_{3} z_{it} + \gamma_{t} + \alpha_{i} + e_{it}$$
- Two stage least square estimator models
  $$y_{it} = \beta_{0} + \beta_{1} \hat{x}_{it} + \beta_{2} z_{it} + \gamma_{t} + \alpha_{i} + e_{it}$$
  $$\hat{x}_{it} = \delta_{0} + \delta_{1} i_{it} + \delta_{2} z_{it} + \psi_{t} + \phi_{i} + \upsilon_{it}$$
- System generalized method of moments estimator (Blundell/Bond [56] and Arellano/Bover [57]) models
  $${\text{Model}}:{ }y_{it} = \beta_{0} + \beta_{1} x_{it - 1} + \beta_{2} y_{it - 1} + \beta_{3} z_{it} + \gamma_{t} + \alpha_{i} + e_{it}$$
  $${\text{Difference}}:{ }y_{it} - y_{it - 1} = \beta_{1} \left( {x_{it - 1} - x_{it - 2} } \right) + \beta_{2} \left( {y_{it - 1} - y_{it - 2} } \right) + \beta_{3} \left( {z_{it} - z_{it - 1} } \right) + \left( {e_{it} - e_{it - 1} } \right)$$
  $${\text{Level}}:{ }y_{it} = \beta_{0} + \beta_{1} x_{it - 1} + \beta_{2} y_{it - 1} + \beta_{3} z_{it - 1} + \gamma_{t} + \alpha_{i} + e_{it}$$
  where $y_{it}$ = vector of dependent variable, $x_{it}$ = vector of independent variables, $\overline{x}_{i}$ = vector of cluster means independent variables, $\hat{x}_{it}$ = vector of estimated independent variables from the first stage least square regression, $z_{it}$ = vector of (time constant) independent control variables, $\beta$ = vector of model coefficients, $\gamma_{t}$ = unobserved time-specific effect, $\alpha_{i}$ = unobserved department-specific effect, $e_{it}$ = individual error term, $\delta$ = vector of parameters to be estimated in the first stage least square regression, $i_{it}$ = instrument for independent variable, $\psi_{t}$ = unobserved time-specific effects in the first stage, $\phi_{i}$ = unobserved department-specific effects in the first stage, $\upsilon_{it}$ = error term in the first stage.

Where entities (departments) are denoted as $i = 1, \ldots ,n$ and observation periods (years) $t = 2015, \ldots , 2019$. The term $\gamma_{t}$ incorporated the time effect on the dependent variable, independent of observable or unobservable differences between individual observation units. This temporal effect captured the influence of changes over time on the dependent variable and served as a global temporal component. $\alpha_{i}$ was the fixed unobserved heterogeneity of each hospital department, and $e_{it}$ signified the error term for each hospital department over time. Standard errors robust for group-wise heteroscedasticity and serial correlation were used. To choose between fixed effects or random effects models, we utilized a robust Hausman-like test.

Organizational characteristics in the German hospital sector exhibit minimal variation and remain relatively time-persistent. Including them in a fixed effect model would render the results valid only in the rare event of a shift in one of the categories, making it unsuitable for a true comparison between different organizational factors. Consequently, alongside a model with fixed effect estimators, we adopted a correlated random effects (CRE) modeling approach [58] to incorporate and control for other organizational variables, enhancing model sensitivity and specificity. Acknowledging potential unmeasured confounders and endogeneity with the explanatory variables in our panel regression models, two models with instrument variables (IV) were constructed and analyzed to address possible endogeneity.

While the panel structure of the data already accounted for some aspects of endogeneity [59], models with instrumental variable estimation were employed to establish potential causal effects. Recommended best practices advocate for the inclusion of additional data to address endogeneity before resorting to IV estimation [58–61], our data source had limitations in providing meaningful potential additional variables. The first model employed a static IV approach with a two-stage least squares (2SLS) estimator, using the number of nursing staff per 1000 deliveries as an external IV. The second model employed a dynamic approach with generalized method of moments (GMM) estimators, incorporating the lagged ratios of observed to expected rates of cesarean births as an internal IV [56, 57, 62]. Additionally, to minimize data loss due to the unbalanced dataset and use more instruments for more efficient estimators, we referred to the system GMM estimator instead a difference GMM estimator [56]. Two-step GMM estimators were chosen for their robustness to autocorrelation and heteroscedasticity [63].

### Statistical analysis

Data preparation (tidyverse package [2.0.0]) and analysis (plm package [2.6–3], lmtest package [0.9–40], gtsummary package [1.7.2], modelsummary package [1.4.3]) were performed in R (version 4.2.2) and R Studio (version 2023.06.1 + 524).

## Results

### Data inclusion

**Fig. 1:**
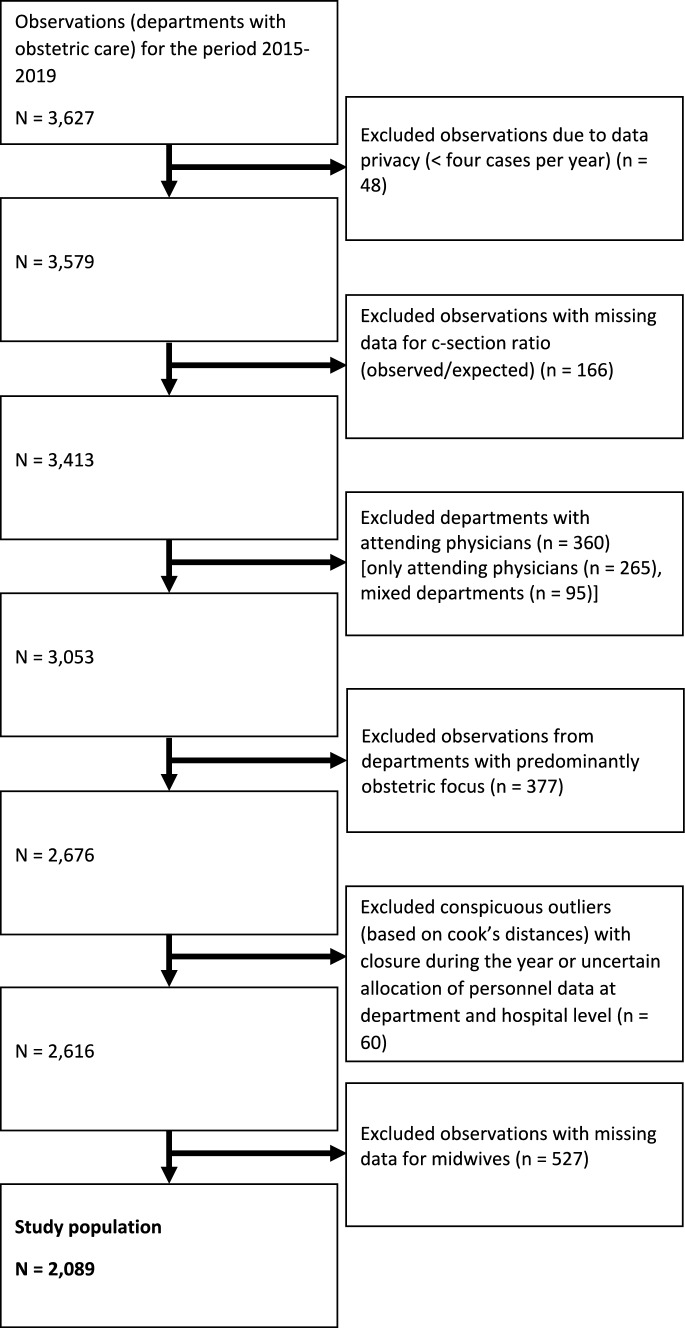
Flow chart of study population

Following the exclusion of duplicate entries, we identified data from 912 departments performing obstetric care, providing 3627 observations from the quality reports (Fig. 1). Regulatory authorities censored 48 observations due to data privacy concerns (less than four deliveries in the reporting year), these were excluded from further analysis. Additionally, 166 observations lacked data on the C-section ratio quality indicator for the respective year, leading to their exclusion. Of the remaining data, 360 reported having attending physicians in the respective year, and these departments were excluded. Similarly, departments primarily offering obstetric services were excluded, with a data-driven cut-off value set at 1.4 times the number of full inpatient cases compared to births (Appendix Fig. 2). 377 observations fell below the threshold and were categorized as solo obstetric departments, subsequently excluded. A manual check using Cook's distance method revealed 60 entries with conspicuous data, confirmed through cross-checking with media reports indicating closures within the reporting year. These departments were consequently excluded. Lastly, 527 observations lacked data on the variable for the number of midwives, resulting in their exclusion. The final analysis included 2089 observations from 519 obstetric departments.

In total, the study population with 2089 observations represented 2,335,839 mothers giving birth and 720,795 C-section deliveries (Table 1). The panel is unbalanced. As the study population no longer corresponded to a full survey of German obstetric departments, we validated the study population against data from IQTIG for the overall numbers on births and cesarean deliveries in obstetric departments in Germany. Over the period 2015 to 2019, the total number of mothers giving births increased from 713,563 to 745,941, with a slight decrease in the C-section rate from 31.42 to 30.85%. These trends were mirrored in the smaller study population (444,555–479,176 births). The study population covered 62.8% of all delivering mothers and 62.4% of all cesarean deliveries in Germany. While the year-by-year C-section rate in the study population was slightly lower than the national rate for all years except 2015, the differences were not significant (t test, p = 0.408). Notably, the deviation in 2016 was attributed to missing data in the quality reports, discussed in more detail in the limitations section. Additionally, solo obstetric departments reported on average cesarean rates (30.3%) similar to those of integrated departments (Appendix Table 8).

**Table 1:** Comparison of births and cesarean sections between total German hospital population and study population

	IQTIG			Study population		
	Deliveries (all mothers who have had at least one birth of a child)	Cesarean deliveries	C-section rate	Deliveries (all mothers who have had at least one birth of a child)	Cesarean deliveries	C-section rate
2015	713,563	224,197	31.42	444,555	139,940	31.48
2016	753,289	235,096	31.21	420,158	130,253	31.00
2017	756,146	235,765	31.18	492,692	151,074	30.66
2018	749,024	229,676	30.66	499,258	152,461	30.54
2019	745,941	230,105	30.85	479,176	147,067	30.69
Σ/Ø	3,717,963	1,154,839	31.06	2,335,839	720,795	30.87

### Characteristics of study population

The observed risk-adjusted C-sections, on average, were marginally below the expected number, indicating a ratio of 0.98 (median 1.00) (Table 2). The number of deliveries per department averaged 1118.2 births. Notably, from 2015 to 2019, the mean number of deliveries exhibited an increase of 123.1, rising from 1031.5 to 1154.6 births, with an intermediate spike observed from 2015 to 2016. However, the median, standing at 913, consistently trailed the mean. Regarding cesarean births, the annual average per department was 345, showing a modest increase of 21.7 from 2015 to 2016. Yet again, the median, at 240.3 cesarean births, lagged behind the mean.

**Table 2:** Descriptive description study population

Characteristic	Overall, N = 2089^1^	2015, N = 431^1^	2016, N = 376^1^	2017, N = 430^1^	2018, N = 437^1^	2019, N = 415^1^
Risk-adjusted cesarean ratio	0.98/1.00 (0.18)	0.97/0.97 (0.19)	0.98/0.99 (0.18)	0.98/1.00 (0.19)	0.99/1.00 (0.18)	0.99/1.01 (0.18)
Number of deliveries	1118.16/913.00 (695.53)	1031.45/828.00 (641.20)	1117.44/905.50 (701.29)	1145.80/934.00 (708.85)	1142.47/936.00 (702.03)	1154.64/950.00 (719.08)
Number of C-sections	345.04/263.00 (240.27)	324.69/250.00 (229.90)	346.42/267.50 (240.73)	351.33/266.00 (241.12)	348.88/265.00 (242.04)	354.38/265.00 (247.48)
Number of full-time equivalent physicians	12.48/10.86 (7.10)	11.75/10.33 (6.57)	12.35/10.73 (7.30)	12.56/11.09 (7.12)	12.74/11.00 (7.17)	13.01/11.14 (7.32)
Number of full-time equivalent physicians per 1000 deliveries	12.46/11.75 (4.45)	12.85/11.84 (4.90)	12.32/11.64 (4.54)	12.22/11.50 (4.36)	12.40/11.84 (4.21)	12.51/11.81 (4.17)
Number of full-time equivalent midwives	11.03/9.17 (7.56)	10.80/9.00 (6.80)	11.11/9.40 (7.56)	11.15/9.16 (7.59)	10.95/9.04 (7.71)	11.16/9.48 (8.13)
Number of full-time equivalent midwives per 1000 deliveries	10.64/10.30 (4.53)	11.38/10.67 (4.35)	10.60/10.14 (4.09)	10.53/10.22 (4.86)	10.25/10.16 (4.31)	10.45/10.37 (4.88)
Number of full-time equivalent nursing staff	23.97/19.60 (17.03)	23.33/19.80 (15.86)	24.60/19.70 (18.54)	24.30/19.65 (16.97)	24.00/19.68 (17.12)	23.64/19.30 (16.68)
Missing	49	40	2	2	3	2
Number of full-time equivalent nursing staff per 1000 deliveries	23.34/21.06 (11.04)	24.69/22.25 (12.18)	23.99/21.90 (11.63)	23.19/21.17 (10.75)	22.76/20.54 (10.20)	22.27/20.12 (10.38)
Missing	49	40	1	2	3	2
*Ownership*						
Non-profit	759 (36.33%)	166 (38.52%)	123 (32.71%)	165 (38.37%)	159 (36.38%)	146 (35.18%)
Private	346 (16.56%)	72 (16.71%)	67 (17.82%)	67 (15.58%)	72 (16.48%)	68 (16.39%)
Public	984 (47.10%)	193 (44.78%)	186 (49.47%)	198 (46.05%)	206 (47.14%)	201 (48.43%)
*Teaching status*						
No teaching assignment	459 (21.97%)	97 (22.51%)	90 (23.94%)	98 (22.79%)	89 (20.37%)	85 (20.48%)
Academic teaching hospital	1472 (70.46%)	309 (71.69%)	256 (68.09%)	298 (69.30%)	313 (71.62%)	296 (71.33%)
University hospital	158 (7.56%)	25 (5.80%)	30 (7.98%)	34 (7.91%)	35 (8.01%)	34 (8.19%)
*Perinatal care level*						
Regular obstetric department (care level 4)	939 (44.95%)	201 (46.64%)	164 (43.62%)	193 (44.88%)	195 (44.62%)	186 (44.82%)
Perinatal focus (care level 3)	405 (19.39%)	73 (16.94%)	73 (19.41%)	87 (20.23%)	90 (20.59%)	82 (19.76%)
Perinatal centers level II (care level 2)	185 (8.86%)	44 (10.21%)	36 (9.57%)	36 (8.37%)	36 (8.24%)	33 (7.95%)
Perinatal centers level I (care level 1)	560 (26.81%)	113 (26.22%)	103 (27.39%)	114 (26.51%)	116 (26.54%)	114 (27.47%)

^a^n (%); mean/median (SD)

The average number of physicians per department over the observation period was 12.5, with a median of 10.9. This represents an increase of more than one full-time equivalent from 2015 to 2019. As outlined earlier, the total number of full-time equivalent physicians was divided by the department's number of births to ensure comparability across departments. The average number of full-time equivalent physicians per 1000 deliveries per department remained constant around 12.5, with a median of 11.8, showing no significant change from 2015 to 2019. In terms of midwives, the average number per department hovered around 11 during the observation period, with a median of 9.2. The average number of full-time equivalent midwives per 1000 deliveries per department was 10.7 (median 10.3). However, from 2015 to 2019, average number of full-time equivalent midwives per 1000 deliveries decreased by nearly one. The average number of full-time equivalent nursing staff (excluding midwives) per department was 24, with a median of 19.6. Per 1000 deliveries, there were 23.3 full-time equivalent nursing staff, with a median of 21.1. Notably, there was a decrease in the average number of full-time equivalent nursing staff per 1000 births from 24.7 in 2015 to 22.3 in 2019.

Private hospitals constituted the minority in the study population at 16.6%, while non-profit hospitals (36.3%) and public hospitals (47.1%) comprised the majority. Regarding academic teaching, 70.5% of hospitals were listed as academic teaching hospitals, 7.6% were university hospitals, and 22% were not engaged in academic teaching. Beyond regular obstetric departments, the landscape encompasses facilities specializing in perinatal care, including level 1 and 2 perinatal centers. Additionally, 45% categorized as departments with the regular level of perinatal care. 19.4% reported a perinatal focus, 8.9% were a level II perinatal center, and 26.8% were a level I perinatal center—the highest form of obstetric health services for high intense care.

Compared to integrated obstetric departments, solo departments, on average, have a significantly lower risk-adjusted cesarean section ratio (0.95; t test, p = 0.001), more deliveries (1563.5; t test, p < 0.001), and more cesarean sections (473.7; t test, p < 0.001), but fewer full-time equivalent physicians (9.7; 6.8 per 1000 deliveries; t test, p < 0.001) and more midwives (13.4; 9.1 per 1000 deliveries; t test, p < 0.001). The crude cesarean section rate did not differ significantly from each other (mean solo department: 30.04%, mean integrated department: 30.41%; t test: p value = 0.371). Solo departments were more likely to be involved in academic teaching (80.1%) and to have a higher perinatal care level (care level 4 = 33.1%; care level 1 = 41.2%) (Appendix Table 9 for more details).

### Uni- and multivariable panel models analyses

Initially, we constructed three univariable models to investigate the impact of physicians per 1000 deliveries, midwives per 1000 deliveries, and total number of 1000 deliveries on the observed-to-expected rates of cesarean delivery (Table 3). All models considered the panel structure of the data, employing fixed-effects estimators that considered both time and individual effects. In the first model, the estimator for the number of physicians per 1000 deliveries demonstrated a significant positive correlation with the observed-to-expected cesarean delivery ratio (0.005; p < 0.001). The second model, focusing on the number of midwives per 1000 deliveries, revealed no significant effect on the ratio of observed-to-expected cesarean births. However, in the third model, the estimator for the number of deliveries per 1000 was significantly negatively correlated with the observed-to-expected cesarean delivery ratio (− 0.057; p = 0.007).

**Table 3:** Uni- and multivariate panel models with two-way fixed effects

	Ratio of observed to expected (O/E) cesarean births (2015–2019) (two-way fixed effects model)			
	Univariate models	Univariate models	Univariate models	Multivariate models
*Number of physicians per 1000 deliveries*	0.005***			0.004**
p value	(< 0.001)			(0.004)
95% CI	[0.002, 0.008]			[0.001, 0.007]
SE	(0.001)			(0.001)
*Number of midwives per 1000 deliveries*		0.002^+^		0.001
p value		(0.079)		(0.375)
95% CI		[0.000, 0.004]		[− 0.001, 0.003]
SE		(0.001)		(0.001)
*Number of deliveries per 1000*			− 0.057**	− 0.016
p value			(0.007)	(0.483)
95% CI			[− 0.098, − 0.016]	[− 0.062, 0.029]
SE			(0.021)	(0.023)
Num. obs	2089	2089	2089	2089
R^2^	0.011	0.003	0.004	0.013
R^2^ adj	− 0.319	− 0.330	− 0.328	− 0.319
AIC	− 5355.2	− 5338.4	− 5340.5	− 5354.3
BIC	− 5343.9	− 5327.1	− 5329.2	− 5331.7
Std. errors	HC1	HC1	HC1	HC1

^+^p < 0.1, *p < 0.05, **p < 0.01, ***p < 0.001

In the pooled multivariate model, no signs of multicollinearity were detected (VIF < 2 for each variable). Subsequently, we developed a multivariate model incorporating all three variables of interest. In this model, only the estimator for the number of physicians per 1000 deliveries exhibited a statistically significant association with the observed-to-expected cesarean delivery ratio (0.004, p = 0.004). A robust Durbin–Wu–Hausman-like test, conducted for heteroscedastic robust standard errors, suggested using models with fixed effects instead of random effects (see Appendix Table 10 for models with random effects estimators) for the univariate model for physicians (p = 0.048) and the multivariate model (p = 0.024), but not for univariate models for midwifes (p = 0.473) and deliveries (p = 0.669).

Overall, this initial analysis revealed a significant effect for the estimator on physicians per birth regarding the observed-to-expected cesarean delivery ratio, although the explanatory power on the C-section ratio was modest. The multivariate model demonstrated a higher within-R^2^ (0.013) compared to the univariate model with only the physicians per birth estimator (R^2^ = 0.011). Additionally, the multivariate model fit the data slightly better (AIC univariate model − 5355.2 to AIC multivariate model − 5354.3).

### Correlated random effects panel model analyses

To incorporate organizational characteristics, we conducted a correlated random effects (CRE) model (Table 4). The three variables from the fixed-effects model exhibited similar effects in the CRE model. The impact of the number of physicians per 1000 deliveries remained consistent (0.005, p = 0.002). According to the robust Hausman tests above, a Wald test again indicated that the random effects assumption was not valid for both models (χ^2^ = 3.21, p = 0.025; χ^2^ = 3.15, p = 0.027). Notably, the department-mean number of physicians per 1000 deliveries, capturing the contextual effect of the variable, showed a significant positive relationship (0.006, p = 0.020). This indicated that if the mean number of physicians per 1000 deliveries increased across integrated obstetric departments, the ratio of observed to expected C-sections also increased. Among the control variables, perinatal centers of the first level, compared to regular obstetric wards, exhibited significantly higher C-section ratios (0.069, p = 0.006). The explained variance increased from 0.074 to 0.104 for the model with organizational controls.

**Table 4:** Multivariate panel models with two-way correlated random estimators

	Ratio of observed to expected (O/E) cesarean births (2015–2019) (two-way fixed effects model)	
	CRE/mixed effects model	CRE/mixed effects model with control variables
*Intercept*	0.886***	0.934***
p value	(< 0.001)	(< 0.001)
95% CI	[0.812, 0.959]	[0.842, 1.025]
SE	(0.038)	(0.047)
*Number of physicians per 1000 deliveries*	0.005**	0.005**
p value	(0.002)	(0.002)
95% CI	[0.002, 0.008]	[0.002, 0.007]
SE	(0.001)	(0.001)
*Mean number of physicians per 1000 deliveries*	0.007**	0.006*
p value	(0.004)	(0.020)
95% CI	[0.002, 0.012]	[0.001, 0.011]
SE	(0.002)	(0.003)
*Number of midwives per 1000 deliveries*	0.001	0.001
p value	(0.352)	(0.310)
95% CI	[− 0.001, 0.004]	[− 0.001, 0.004]
SE	(0.001)	(0.001)
*Mean number of midwives per 1000 deliveries*	− 0.004^+^	− 0.004^+^
p value	(0.094)	(0.087)
95% CI	[− 0.009, 0.001]	[− 0.009, 0.001]
SE	(0.003)	(0.003)
*Number of deliveries per 1000*	− 0.003	− 0.009
p value	(0.882)	(0.698)
95% CI	[− 0.046, 0.040]	[− 0.052, 0.035]
SE	(0.022)	(0.022)
*Mean number of deliveries per 1000*	− 0.008	− 0.037
p value	(0.752)	(0.159)
95% CI	[− 0.057, 0.041]	[− 0.088, 0.014]
SE	(0.025)	(0.026)
*Ownership: public (ref. category: ownership private)*		− 0.033
p value		(0.164)
95% CI		[− 0.079, 0.013]
SE		(0.024)
*Ownership: non-profit (ref. category: ownership private)*		− 0.035
p value		(0.151)
95% CI		[− 0.082, 0.013]
SE		(0.024)
*Perinatal centers level I (care level 1) (ref. category: regular obstetric department (care level 4))*		0.069**
p value		(0.006)
95% CI		[0.019, 0.118]
SE		(0.025)
*Perinatal centers level II (care level 2) (ref. category: regular obstetric department (care level 4)*		0.012
p value		(0.650)
95% CI		[− 0.040, 0.065]
SE		(0.027)
*Perinatal focus (care level 3) (ref. category: regular obstetric department (care level 4)*		0.001
p value		(0.978)
95% CI		[− 0.042, 0.043]
SE		(0.022)
*Teaching status: academic teaching hospital (ref. category: teaching status: no teaching assignment)*		0.014
p value		(0.483)
95% CI		[− 0.025, 0.052]
SE		(0.020)
*Teaching status: University Hospital (ref. category: teaching status: no teaching assignment)*		0.017
p value		(0.622)
95% CI		[− 0.050, 0.083]
SE		(0.034)
Num. obs	2089	2089
R^2^	0.074	0.104
R^2^ adj	0.071	0.099
AIC	− 1310.8	− 1367.2
BIC	− 1265.7	− 1282.6

^+^p < 0.1, *p < 0.05, **p < 0.01, ***p < 0.001

## Instrument variable analyses

### Two stage least squares estimator

For the first instrumental variable analysis, we employed the number of nursing staff (excluding midwives) per 1000 deliveries as the IV for the number of physicians per 1000 deliveries. We excluded departments with missing values on nursing staff (n = 11) and outliers (n = 3). Additionally, departments from one German private hospital group were excluded for the year 2015 (n = 38) since these hospitals provided staffing numbers on nursing staff at the hospital rather than the department level. Consequently, our analysis incorporated 2037 observations from 516 departments.

#### Relevance

The number of nursing staff per 1000 deliveries exhibited a significant positive correlation with the number of physicians per 1000 deliveries (0.239, p < 0.001) (Appendix Table 11). The F-statistic for the model was at 237.2 with robust standard errors. Traditionally, a value above 10 is assumed for the suitability of an instrument variable, but according to Lee, McCrary, Moreira, Porter [64], a value of 104 is considered a better significance threshold.

#### Excludability

While nursing professionals play a crucial role in the care of mothers and newborns within an obstetric department, they lack a direct decision-making role in the choice of the delivery mode. Unlike midwives and physicians, who may influence delivery decisions, nursing staff primarily engage in postnatal care. Additionally, nursing staff in obstetric departments provide guidance on newborn care and support mothers in the postpartum period. We view nursing staff as a reflection of the general staffing situation in the department, with the cesarean section ratio linked to the number of nursing staff solely through the number of physicians.

#### Exogeneity

Even though the IV, explanatory, and outcome variables existed within the same organizational context, the panel structure allowed us to detach time-persistent influences from the analysis. For instance, the perinatal level of care may impact the nursing staff level per birth in the department. Obstetric departments with higher care levels tend to have more nursing staff due to caring for preterm infants or newborns with health issues. Pre-selection occurs here, with hospitals admitting pregnant women at risk to those with a high perinatal care level. Panel estimators controlled for such biases, ensuring the fixed effects estimator's robustness. Influencing time-persistent factors like the organizational culture were also controlled for in the panel structure. In our assessment, nursing staff served as a suitable instrumental variable for physicians and their influence on the mode of delivery.

#### Results

With the employed IV, the effect size of the variable for the number of physicians per 1000 deliveries increased to 0.014 (p < 0.001) in the univariate model (Table 5). This significant effect persisted in the multivariate model (0.016, p < 0.001). Overall, the effect size tripled compared to the fixed-effects and CRE models, and the within-R^2^ increased to 0.060 and 0.059, respectively.

**Table 5:** Static IV models with two stage least square estimators

	Ratio of observed to expected (O/E) cesarean births (2015–2019) (two-way fixed effects model)	
	Univariate model	Multivariate model
*Number of physicians per 1000 deliveries*	0.014***	0.016***
p value	(< 0.001)	(< 0.001)
95% CI	[0.009, 0.019]	[0.009, 0.022]
SE	(0.003)	(0.003)
*Number of midwives per 1000 deliveries*		− 0.002
p value		(0.288)
95% CI		[− 0.005, 0.002]
SE		(0.002)
*Number of deliveries per 1000*		0.016
p value		(0.281)
95% CI		[− 0.013, 0.045]
SE		(0.015)
Num. obs	2037	2037
R^2^	0.060	0.059
R^2^ adj	− 0.253	− 0.256
AIC	− 3399.5	− 3386.4
BIC	− 3388.3	− 3363.9
Std. errors	HC1	HC1

^+^p < 0.1, *p < 0.05, **p < 0.01, ***p < 0.001

The residuals from the instrument variable analysis, encompassing endogenous information, signaled that the number of physicians per 1000 deliveries was indeed endogenous (p < 0.005 for both models) (see Appendix Table 12). Therefore, it appears prudent to employ an appropriate instrumental variable to attain a purely exogenous effect.

#### System generalized method of moments estimator

In a dynamic setting, considering the C-section ratio from past years to control for potential endogeneity, we utilized system generalized method of moments (GMM) estimations. Although a comparison with estimators for the lagged variable from ordinary least squares (OLS) and fixed-effect models (Appendix Table 13) revealed a tendency to favor a difference GMM estimation approach, as the outcome variable did not display strong time persistence. Given the unbalanced panel dataset with a brief time span of only five observation points, we opted for a system GMM approach. The results of the difference GMM model can be found in the appendix (Appendix Table 14).

The univariate model showed a significant effect of the physician variable (0.004, p < 0.001) (Table 6). In the multivariate model, both without and with control variables, the significant effect for the physician variable persisted (0.004, p < 0.001). Furthermore, in the full model with control variables, the variable for the number of births became negatively significant (− 0.037, p < 0.001). Notably, the volume variable attained significance due to its inclusion in the control variable for perinatal care level (not shown in the table). Specifically, the variable for perinatal centers level I displayed a significant positive effect (0.051, p < 0.001). The Hansen-Sargan test indicated a robust non-overfitting of instrumental variables (p > 0.35 for all three models). The autocorrelation test for the second order yielded non-significant results for all three models (p < 0.001). While the Wald test for the coefficients exhibited significant effects of the joint independent and control variables in all three models (p < 0.001), the Wald test for the joint time dummies indicated no significant effect in all three models (p > 0.05).

**Table 6:** Dynamic IV models with system generalized method of moment estimators

	Ratio of observed to expected (O/E) cesarean births (2015–2019) (two-way fixed effects model)		
	Univariate model	Multivariate model	Multivariate model with control variables
*Lagged ratio of observed to expected ratio (O/E) of cesarean births*	0.485***	0.503***	0.489***
p value	(< 0.001)	(< 0.001)	(< 0.001)
95% CI	[0.355, 0.616]	[0.374, 0.633]	[0.383, 0.596]
SE	(0.068)	(0.069)	(0.054)
*Number of physicians per 1000 deliveries*	0.004***	0.004***	0.004**
p value	(< 0.001)	(< 0.001)	(0.002)
95% CI	[0.002, 0.006]	[0.002, 0.007]	[0.001, 0.006]
SE	(0.001)	(0.001)	(0.001)
*Number of midwives per 1000 deliveries*		− 0.002	− 0.002
p value		(0.164)	(0.105)
95% CI		[− 0.004, 0.001]	[− 0.004, 0.000]
SE		(0.001)	(0.001)
*Number of deliveries per 1000*		− 0.010^+^	− 0.037***
p value		(0.095)	(< 0.001)
95% CI		[− 0.022, 0.002]	[− 0.054, − 0.020]
SE		(0.006)	(0.009)
*Ownership: public (ref. category: ownership private)*			− 0.015
p value			(0.225)
95% CI			[− 0.039, 0.009]
SE			(0.013)
*Ownership: non-profit (ref. category: ownership private)*			− 0.002
p value			(0.906)
95% CI			[− 0.027, 0.024]
SE			(0.014)
Perinatal centers level I (care level 1) (ref. category: regular obstetric department (care level 4))			0.051***
p value			(< 0.001)
95% CI			[0.024, 0.078]
SE			(0.014)
*Perinatal centers level II (care level 2) (ref. category: regular obstetric department (care level 4)*			0.016
p value			(0.304)
95% CI			[− 0.014, 0.045]
SE			(0.016)
*Perinatal focus (care level 3) (ref. category: regular obstetric department (care level 4)*			− 0.010
p value			(0.355)
95% CI			[− 0.033, 0.012]
SE			(0.012)
*Teaching status: academic teaching hospital (ref. category: teaching status: no teaching assignment)*			0.013
p value			(0.224)
95% CI			[− 0.008, 0.034]
SE			(0.011)
*Teaching status: University Hospital (ref. category: teaching status: no teaching assignment)*			0.014
p value			(0.263)
95% CI			[− 0.010, 0.037]
SE			(0.013)
Num. obs	2520	2520	2520
Hansen–Sargan test/J test (p value)	6.983 (0.639)	11.725 (0.385)	12.310 (0.831)
Arellano–bond test/autocorrelation test (1) (p value)	− 6.094 (< 0.001)	− 6.058 (< 0.001)	− 6.321 (< 0.001)
Arellano–bond test/autocorrelation test (2) (p value)	− 0.101 (0.919)	− 0.094 (0.925)	− 0.253 (0.800)
Wald test for coefficients (p value)	110.125 (< 0.001)	148.589 (< 0.001)	321.429 (< 0.001)
Wald test for time dummies (p value)	3.130 (0.372)	3.084 (0.379)	4.702 (0.195)
Std. errors	HC1	HC1	HC1

^+^p < 0.1, *p < 0.05, **p < 0.01, ***p < 0.001

#### Outcome validation

We conducted the same analyses for departments primarily focused on obstetric care (n = 307) and found no significant correlations for any variables (Appendix Tables 15 and 16). Additionally, we examined the crude cesarean section ratio (number of cesareans divided by the total number of deliveries) and observed similar positive relationships with the variable for the number of full-time equivalent physicians per 1000 deliveries (Appendix Tables 17, 18, 19). However, some instrumental variable analyses did not reveal a significant effect for the number of physicians per 1000 deliveries (Appendix Tables 20, 21).

## Discussion

Existing literature highlights the global increase in cesarean section rates, often attributed to non-medical factors, including maternal preferences and organizational influences. While previous studies have examined individual and hospital-level factors affecting C-section rates, such as physician characteristics, hospital ownership, and caseload, there is limited focus on the interactions within obstetric departments, particularly regarding medical staffing. In this study, we conducted an initial investigation of the roles played by physicians, midwives, and the number of births and C-section in German combined obstetrics and gynecological departments from 2015 to 2019, encompassing over 60% of births in the country. Our study addressed gaps in previous research by employing a longitudinal approach, controlling for both medical and organizational factors, and accounting for methodological biases related to medical indications.

Across various models and study designs, our findings consistently revealed a significant positive correlation between the number of full-time equivalent physicians per delivery in integrated obstetric and gynecological departments and the risk-adjusted observed-to-expected cesarean delivery ratio. Intriguingly, multiple instrumental variable analyses hinted at a potential causal effect for this relationship. These results challenge the notion that cesarean sections are a preferable management strategy during staff shortages [25]. While our study design does not allow us to pinpoint the cause of this effect, the absence of a significant within-effect in solo departments, along with the significantly lower risk-adjusted cesarean rates in solo departments compared to integrated departments, suggests that specialization may be a contributing factor. This would evidence towards a specialization argument for obstetric care [17].

Noteworthy, other studies have reported an inverse effect for the number of physicians per delivery. For instance, Zibri et al. [36] examined eleven French hospitals from 2008 to 2014, while Gombolay et al. [37] conducted an extensive cross-sectional analysis for 2014 in a tertiary care medical center in Boston. The disparity in hospital populations, national characteristics of hospital structures, and methodological approaches could explain these contradictory findings. Overall, the importance of staffing levels is recognized by physician managers in quality research [31].

Caution is warranted when interpreting the greater effect size for the 2SLS-estimators, as the instrument variable (nursing staff per 1000 deliveries) may introduce bias, potentially overestimating the effect. Additionally, since the system and difference GMM estimators revealed comparable effect size for the number of physicians per delivery on the cesarean section ratio, the difference GMM estimators potentially lack statistical power. Hence, concerns about misleading effects with the system GMM estimator are of minor significance.

The objective of minimizing cesarean sections itself is open to debate. C-sections should be performed when medically necessary or at the request of the woman giving birth. Emphasizing the reduction of C-sections could lead to compromised care quality if a C-section is medically needed but not performed. The fact that the average risk-adjusted cesarean ratio was 0.98—two percent below the expected number of C-sections—suggests that there may already be an underprovision of C-sections in Germany. Conversely, the underperformance might also indicate potential issues with the risk adjustment computation. With the introduction to the Robson indicator from 2020 onwards, risk adjustment will become more internationally comparable. This will allow for a better assessment of whether the original indicator was sufficiently suitable. Overall, among the available quality indicators (Appendix Table 7), we believe that the risk-adjusted cesarean ratio is particularly well-suited for comparing obstetric care quality. Other indicators, such as infant mortality, were either not collected or too rare to be reliable, as in the case of maternal mortality (fewer than 25 cases per year).

Beyond our focus on delivery methods, higher staffing levels have been associated with better quality of care and higher patient satisfaction in obstetrician care [65]. Effective communication between patients and clinicians contributes to reducing high cesarean section rates [66]. Evidence from a Cochrane review suggests that continuous support during labor—one-to-one intrapartum support compared with usual care—is associated with a lower rate of cesarean sections, emphasizing the potential impact of staffing levels on provider-patient communication [67]. Therefore, from this perspective, higher staffing levels in the department should theoretically result in a lower ratio of cesarean sections.

While only a few models established an effect for the number of deliveries when examined together with the number of physicians per birth, other studies have reported such associations [35, 36, 68, 69], while other did not [70]. Additionally, the “practice-makes-perfect hypothesis”, widely observed in health quality research [71, 72] and obstetrics care [53], indicates that hospitals with more births perform fewer cesarean sections. Our results underscore the importance of not solely focusing on the overall number of deliveries within a department. Instead, they underscore the importance of relating the number of deliveries to the number of physicians within the department.

Regarding the number of midwives per births, we could not identify a significant effect of the estimator on the cesarean section ratio. Numerous studies report a correlation between midwifery-led delivery or the volume of midwives in an obstetric department and lower cesarean section rates compared to obstetric care at birth [36, 72–81]. However, other studies did not report such a correlation [79, 82].

In Germany, labor in a hospital is often supervised by a physician, limiting the influence of midwives on the decision of the delivery method compared to other countries. Although midwife-led delivery rooms exist, where midwives perform birth without physician supervision, this approach has been seldom researched [83, 84] and is not separately documented in the quality reports.

### Strengths and limitations

This analysis relies on secondary data obtained from German hospitals, specifically from quality reports not originally intended for research purposes but rather aimed at enhancing transparency in inpatient care. While the study identified a potential impact of the number of physicians on the risk-adjusted C-section ratio, it is important to note that information regarding the processes and distribution of physicians' tasks within combined obstetric and gynecological departments remains unknown.

The use of aggregated panel data at the department level introduces an element of ambiguity in the data on physician staffing. While midwives are reasonably assumed to be primarily engaged in obstetric care, the clarity of physician allocation between obstetric and gynecological care is less apparent. However, assuming a consistent situation over time within each department aids in mitigating the potential ambiguity inherent in the study population. Nevertheless, the identified patterns necessitate further investigation into the workflow of combined obstetric and gynecological departments, and the possibility of an omitted variable bias cannot be ruled out [85].

Additionally, the absence of details on physician characteristics in the data is a limitation. Research indicates that gender may influence cesarean delivery rates, with female physicians performing fewer cesarean deliveries and exhibiting a lower preference for them [86]. While male physician tend to perform non-medically indicated cesarean section (relative indications) on maternal request more willingly than their female counter parts [81]. Our results remain valid under the assumption that the distribution of physicians by gender or other influential factors at the physician level did not systematically vary across departments throughout the study period.

Furthermore, the results of the analysis hold true if no systematic changes in the socio-cultural composition of the patient population and no systematic changes in patient requests for cesarean sections occurred within the study period.

Workload variations between working days and weekends, as well as day and night shifts, may impact medical treatment decisions and quality aspects [87]. Although annual average data were employed, the assumption that these aspects vary randomly between departments is crucial for the presented results to hold true.

Low explanatory power is a common issue in volume-based studies [88] and within-department analyses in obstetric care [22]. For static IV analyses using nursing staff as an instrument for physician staffing, joint confounders on all variables could not be ruled out, given that all variables originated from the same organization. The panel structure, while beneficial in isolating time- and department-specific influences, may not eliminate all influencing factors that could account for the higher observed effect size in the 2SLS models.

In the data for 2016, there was a noticeable reduction in the number of departments. This discrepancy was evident in other study at the topic as well [18]. Upon closer examination of the data, it became apparent that the absent departments were predominantly from the federal state of North Rhine-Westphalia. Specifically, we identified more than 70 departments that were present in the data for both 2015 and 2017 but conspicuously absent in 2016. Despite efforts to address this issue through consultation with the regulatory authorities (IQTIG), obtaining these missing data retroactively or generating them from other sources within the quality reports proved unfeasible. Consequently, the data for the year 2016 contain a bias due to the absence of these departments.

Remarkably, a considerable number of missing values pertaining to midwives were noted, despite regulatory mandates requiring their presence at all hospital births in Germany. The imperative for a midwife's attendance extends to all hospital births, including cesarean sections (§ 4 Abs 1.-Hebammengesetz). However, certain departments reported the presence of attending midwives, leading to the exclusion from the study. A notable percentage of hospitals indicated either a complete absence of midwives or reported them outside obstetric departments. A recurring observation was that departments slated for closure in the subsequent year failed to furnish any data on medical staff for the preceding year. This phenomenon can be attributed to the time lag of over a year in the data collection and compilation process. The overall data quality in this aspect is suboptimal, potentially serving as a source of bias.

Finally, it is crucial to recognize that healthcare, particularly obstetric care, is significantly influenced by cultural, policy, and local factors [8, 33, 89, 90]. The complex and divergent nature of the relationship between these factors underscores the challenge of drawing general and cross-national conclusions from the study results [37].

### Implications for practice

The findings underscore that within integrated gynecological and obstetric departments, a higher number of physicians per delivery significantly correlated with more observed risk-adjusted C-sections than expected. The dual provision of gynecological and obstetric care in one department poses a challenge, potentially contributing to an elevated C-section ratio with an increased number of physicians per birth as each physician may be less acquainted with obstetric care.

As a practical approach, directing attention towards focus and specialization within the medical team could yield a reduction in the C-section ratio, potentially involving intra-department staff reallocation allowing for a more specialized workforce between the two areas of responsibility. This approach does not necessarily imply reducing the total number of physicians but rather enhancing specialization between obstetrics and gynecology care.

An insightful analysis of departments surpassing the reference value (90th percentile) reveals that these departments (n = 118) exhibit an average of 15.1 physicians per 1000 deliveries, accompanied by 872.5 births and 358.9 C-sections (41.1%). In contrast, inconspicuous departments (n = 1971) exhibit an average of 12.3 physicians per 1000 deliveries, along with 1132.9 births and 344.2 C-sections (30.4%). A targeted reduction of physicians per 1000 births by 3, aligning with the inconspicuous department average, could anticipate a modest reduction of 0.012 in the observed expected C-sections, based on our results. On the other hand, departments in the 10th percentile (n = 217) deployed on average 10.9 physicians per 1000 deliveries, accompanied by 1216.5 births and 259.7 C-sections (21.3%). Importantly, as we estimated within-effects the effect size is smaller as a between comparison would suggest as they incorporate department individual effects. As for departments that already have low C-section rates (and a potential underperformance of C-sections) it is unclear whether a further increase in the number of deliveries per physician would lead to improved quality of care.

These implications suggest that a nuanced approach to physician staffing, coupled with a strategic focus on specialization and a potential realignment of resources, could contribute to achieve the objective of the quality indicator of minimizing C-sections within integrated obstetric and gynecological departments. If the goal, as articulated in the quality indicator, is to minimize the occurrence of cesarean section births, it is essential to consider additional factors beyond the number of physicians per birth presented in this analysis. The assessment must also consider the preferences of the woman giving birth and relevant medical considerations, ensuring that these crucial aspects are not disregarded. It is vital that a narrow focus on reducing the cesarean section rate does not compromise the health of women and infants.

## Conclusion

Our examination of integrated German obstetric and gynecological departments revealed a noteworthy and robust positive association between the number of physicians per birth and an elevated ratio of observed-to-expected cesarean sections. Additionally, instrumental variable analysis indicated a potential causal effect. However, given the reliance on annual, aggregated averages and the inherent uncertainties despite the instrumental analysis employed, a cautious approach is essential when interpreting causality. Preliminary interpretations suggest that specialization, as indicated by the number of deliveries per physician, may influences the cesarean section ratio within integrated obstetric and gynecological departments.

## Appendix

**Fig. 2:**
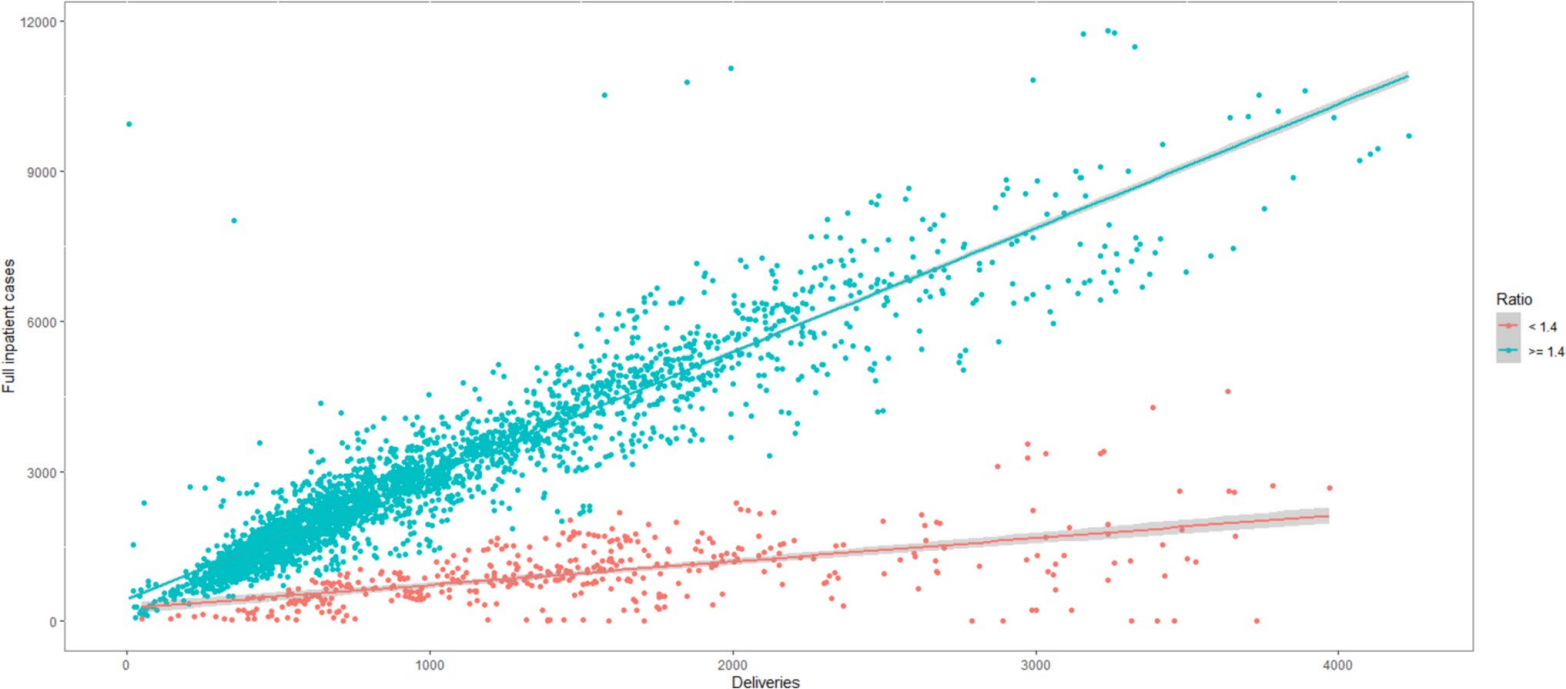
Ratio deliveries to full inpatient cases

See Tables 6, 7, 8, 9, 10, 11, 12, 13, 14, 15, 16, 17, 18, 19, 20, 21, 22 and Fig. 2.

**Table 7:** Obstetric quality indicators for the period 2015–2019

QI-ID	Indicator description	Years	Quality goal
330	Antenatal corticosteroid therapy for preterm births with a prepartum inpatient stay of at least two calendar days	2015–2019	Frequent initiation of antenatal corticosteroid therapy (lung maturation induction) in births with a gestational age of 24 + 0 to under 34 + 0 weeks, excluding stillbirths, and with a prepartum inpatient stay of at least two calendar days
50,046	Administration of antibiotics in cases of premature rupture of membranes	2015	Not listed in 2015
50,045	Perioperative antibiotic prophylaxis in cesarean section deliveries	2015–2019	High rate of perioperative antibiotic prophylaxis in cesarean section deliveries
52,243	Cesarean births	2015	Not listed in 2015
52,249	Ratio of observed to expected rate (O/E) of cesarean births	2015–2019	Low rate of cesarean births
1058	Decision-to-delivery interval over 20 min in emergency cesarean sections	2015–2019	Rarely a decision-to-delivery interval of more than 20 min in emergency cesarean sections
319	Measurement of umbilical artery pH in singleton live births	2015	Not listed in 2015
321	Acidosis in mature singletons with umbilical artery pH measurement	2015–2019	Low rate of acidosis in singleton live births with umbilical artery pH measurement
51,397	Ratio of observed to expected rate (O/E) of acidosis in mature singletons with umbilical artery pH measurement	2015–2019	Low rate of acidosis in singleton live births with umbilical artery pH measurement
51,826	Acidosis in preterm singletons with umbilical artery pH measurement	2015	Not listed in 2015
51,831	Ratio of observed to expected rate (O/E) of acidosis in preterm singletons with umbilical artery pH measurement	2015–2019	Low rate of acidosis in singleton live births with umbilical artery pH measurement
318	Presence of a pediatrician at preterm births	2015–2019	Frequent presence of a pediatrician at the birth of preterm live births with a gestational age of 24 + 0 to under 35 + 0 weeks
1059	Critical outcome in mature newborns	2015	Not listed in 2015
51,803	Quality index for critical outcome in mature newborns	2015–2019	Rare occurrences of child deaths, 5-min Apgar score below 5, pH below 7, and Base Excess < − 16 in mature newborns
51808_51803	Level 1: Ratio of observed to expected rate (O/E) of child deaths	2018, 2019	Not specified
51813_51803	Level 2: Ratio of observed to expected rate (O/E) of children with a 5-min Apgar score below 5	2018, 2019	Not specified
51818_51803	Level 3: Ratio of observed to expected rate (O/E) of children with Base Excess below − 16	2018, 2019	Not specified
51823_51803	Level 4: Ratio of observed to expected rate (O/E) of children with acidosis (pH < 7.00)	2018, 2019	Not specified
322	Third- or fourth-degree perineal tear in spontaneous singleton deliveries	2015	Not specified
51,181	Ratio of observed to expected rate (O/E) of third- or fourth-degree perineal tears in spontaneous singleton deliveries	2015–2017	Low number of mothers with third- or fourth-degree perineal tears in spontaneous singleton deliveries
323	Third- or fourth-degree perineal tear in spontaneous singleton deliveries without episiotomy	2015	Not listed in 2015
324	Third- or fourth-degree perineal tear in spontaneous singleton deliveries with episiotomy	2015	Not listed in 2015
52,244	Mothers and children discharged home together	2015	Not listed in 2015
52,254	Ratio of observed to expected rate (O/E) of mothers and children discharged home together	2015	Not listed in 2015
331	Maternal mortality in the context of perinatal surveys	2015–2019	Rare occurrences of maternal deaths
181,800	Quality index for fourth-degree perineal tears in singleton deliveries	2018, 2019	Low number of mothers with fourth-degree perineal tears in spontaneous or vaginal-assisted singleton deliveries
181801_181800	Level 1: Ratio of observed to expected rate (O/E) of fourth-degree perineal tears in spontaneous singleton deliveries	2018, 2019	Not specified
181802_181800	Level 2: Ratio of observed to expected rate (O/E) of fourth-degree perineal tears in vaginal-assisted singleton deliveries	2018, 2019	Not specified

**Table 8:** Comparison of births and cesarean sections between integrated and solo obstetric departments

	Study population (integrated departments)			Solo departments		
	Deliveries (all mothers who have had at least one birth of a child)	Cesarean deliveries	C-section rate	Deliveries (all mothers who have had at least one birth of a child)	Cesarean deliveries	C-section rate
2015	444,555	139,940	31.48	90,690	28,866	31.83
2016	420,158	130,253	31.00	93,824	27,489	29.30
2017	492,692	151,074	30.66	91,709	28,149	30.69
2018	499,258	152,461	30.54	98,282	28,996	29.50
2019	479,176	147,067	30.69	107,058	32,398	30.26
Σ/Ø	2,335,839	720,795	30.87	481,563	145,898	30.32

**Table 9:** Descriptive description solo obstetric departments

Characteristic	Overall N = 308^a^	2015 N = 61^a^	2016 N = 57^a^	2017 N = 61^a^	2018 N = 62^a^	2019 N = 67^a^
Risk-adjusted cesarean ratio	0.95/0.95 (0.18)	0.97/0.99 (0.17)	0.92/0.92 (0.19)	0.95/0.96 (0.18)	0.94/0.94 (0.18)	0.95/0.96 (0.19)
Number of deliveries	1563.52/1474.50 (709.62)	1486.72/1293.00 (716.81)	1646.04/1603.00 (785.32)	1503.43/1464.00 (634.92)	1585.19/1523.00 (681.82)	1597.88/1520.00 (734.48)
Number of C-sections	473.69/437.00 (249.22)	473.21/436.00 (253.57)	482.26/437.00 (282.01)	461.46/433.00 (225.98)	467.68/426.50 (229.55)	483.55/448.00 (259.85)
Number of full-time equivalent physicians	9.66/8.34 (4.96)	9.28/8.25 (5.03)	9.73/8.60 (5.59)	9.19/8.04 (4.22)	9.74/8.56 (4.56)	https://doi.org/10.32/8.34 (5.36)
Number of full-time equivalent physicians per 1000 deliveries	6.82/5.82 (3.31)	7.03/6.08 (3.68)	6.72/5.79 (3.52)	6.79/5.48 (3.33)	6.68/5.70 (3.03)	6.89/6.16 (3.09)
Number of full-time equivalent midwives	13.39/12.79 (7.38)	13.22/12.30 (8.20)	13.72/12.80 (8.02)	12.61/12.64 (6.77)	13.24/12.89 (6.85)	14.12/13.60 (7.17)
Number of full-time equivalent midwives per 1000 deliveries	9.07/9.20 (3.80)	9.57/9.19 (4.84)	8.95/8.86 (4.14)	8.70/9.29 (3.24)	8.79/9.08 (3.23)	9.32/9.52 (3.41)
Number of full-time equivalent nursing staff	17.65/15.88 (9.93)	18.05/16.00 (9.89)	18.81/15.80 (12.93)	16.39/15.14 (7.99)	17.97/16.66 (10.31)	17.13/16.19 (8.26)
missing	1	0	0	0	0	1
Number of full-time equivalent nursing staff per 1000 deliveries	12.17/10.93 (5.92)	13.51/12.29 (7.67)	12.23/10.54 (6.33)	11.41/10.94 (4.42)	12.25/10.58 (6.17)	11.53/11.09 (4.45)
Missing	1	0	0	0	0	1
*Ownership*						
Non-profit	176 (57.14%)	34 (55.74%)	29 (50.88%)	34 (55.74%)	38 (61.29%)	41 (61.19%)
Private	31 (10.06%)	6 (9.84%)	8 (14.04%)	5 (8.20%)	5 (8.06%)	7 (10.45%)
Public	101 (32.79%)	21 (34.43%)	20 (35.09%)	22 (36.07%)	19 (30.65%)	19 (28.36%)
*Teaching status*						
No teaching assignment	39 (12.66%)	8 (13.11%)	6 (10.53%)	10 (16.39%)	9 (14.52%)	6 (8.96%)
Academic teaching hospital	247 (80.19%)	51 (83.61%)	47 (82.46%)	47 (77.05%)	47 (75.81%)	55 (82.09%)
University hospital	22 (7.14%)	2 (3.28%)	4 (7.02%)	4 (6.56%)	6 (9.68%)	6 (8.96%)
*Perinatal care level*						
Regular obstetric department (care level 4)	102 (33.12%)	18 (29.51%)	23 (40.35%)	19 (31.15%)	20 (32.26%)	22 (32.84%)
Perinatal focus (care level 3)	31 (10.06%)	6 (9.84%)	4 (7.02%)	7 (11.48%)	7 (11.29%)	7 (10.45%)
Perinatal centers level II (care level 2)	48 (15.58%)	11 (18.03%)	8 (14.04%)	10 (16.39%)	9 (14.52%)	10 (14.93%)
Perinatal centers level I (care level 1)	127 (41.23%)	26 (42.62%)	22 (38.60%)	25 (40.98%)	26 (41.94%)	28 (41.79%)

^a^n (%); mean/median (SD)

**Table 10:** Uni- and multivariate panel models with two-ways random effects on risk-adjusted C-section ratio

	Ratio of observed to expected (O/E) cesarean births (2015–2019) (two-way random effects model)			
	Univariate models			Multivariate model
*Intercept*	0.893***	0.964***	1.022***	0.918***
*p* value	(< 0.001)	(< 0.001)	(< 0.001)	(< 0.001)
95% CI	[0.852, 0.934]	[0.924, 1.004]	[0.994, 1.051]	[0.852, 0.984]
SE	(0.021)	(0.020)	(0.014)	(0.034)
*Number of physicians per 1000 deliveries*	0.007***			0.007***
p value	(< 0.001)			(< 0.001)
95% CI	[0.004, 0.010]			[0.003, 0.010]
SE	(0.002)			(0.002)
*Number of midwives per 1000 deliveries*		0.002		0.000
p value		(0.327)		(0.912)
95% CI		[− 0.002, 0.005]		[− 0.004, 0.004]
SE		(0.002)		(0.002)
*Number of deliveries per 1000*			− 0.036***	− 0.017
p value			(< 0.001)	(0.135)
95% CI			[− 0.056, − 0.015]	[− 0.039, 0.005]
SE			(0.010)	(0.011)
Num. obs	2089	2089	2089	2089
R^2^	0.061	0.000	0.019	0.059
R^2^ adj	0.061	0.000	0.019	0.057
AIC	− 1282.3	− 1158.3	− 1201.5	− 1279.1
BIC	− 1265.3	− 1141.3	− 1184.5	− 1250.9
Std. errors	HC1	HC1	HC1	HC1

^+^p < 0.1, *p < 0.05, **p < 0.01, ***p < 0.001

**Table 11:** First stage least square regression for number of nursing staff per 1000 deliveries as an instrument variable for number of physicians per 1000 deliveries

	First stage regression (fixed effects)
*Number of nursing staff per 1000. deliveries*	0.239***
p value	(< 0.001)
95% CI	[0.208, 0.269]
SE	(0.016)
Num. obs	2037
R^2^	0.329
R^2^ adj	0.106
AIC	9101.9
BIC	9113.1
F-statistic	237.206
Std. errors	HC1

^+^p < 0.1, * p < 0.05, ** p < 0.01, *** p < 0.001

**Table 12:** Endogeneity check for instrument variable on risk-adjusted C-section ratio

	Ratio of observed to expected (O/E) cesarean births (2015–2019) (two-way fixed effects model)	
	univariate model	multivariate model
*Number of physicians per 1000 deliveries*	0.014***	0.014***
p value	(< 0.001)	(< 0.001)
95% CI	[0.009, 0.019]	[0.009, 0.019]
SE	(0.003)	(0.003)
*Number of midwives per 1000 deliveries*		− 0.001
p value		(0.627)
95% CI		[− 0.004, 0.002]
SE		(0.002)
*Number of deliveries per 1000*		0.001
p value		(0.933)
95% CI		[− 0.024, 0.026]
SE		(0.013)
Endogenous part from the IV	− 0.006*	− 0.006*
p value	(0.023)	(0.021)
95% CI	[− 0.012, − 0.001]	[− 0.012, − 0.001]
SE	(0.003)	(0.003)
Num. obs	2037	2037
R^2^	0.066	0.067
R^2^ adj	− 0.246	− 0.247
AIC	− 3436.6	− 3433.6
BIC	− 3419.7	− 3405.5
Std. errors	HC1	HC1

^+^p < 0.1, *p < 0.05, **p < 0.01, ***p < 0.001

**Table 13:** Comparison of effect size lagged variable for OLS, fixed effect and difference GMM estimator on risk-adjusted C-section ratio

	Ratio of observed to expected (O/E) cesarean births (2015–2019) (two-way fixed effects model)		
	OLS	Panel model with fixed effects	difference GMM
*Intercept*	0.144***		
p value	(< 0.001)		
95% CI	[0.111, 0.177]		
SE	(0.017)		
Lagged ratio of observed to expected ratio (O/E) of cesarean births	0.864***	0.046	0.510***
p value	(< 0.001)	(0.241)	(< 0.001)
95% CI	[0.837, 0.891]	[− 0.031, 0.123]	[0.265, 0.754]
SE	(0.014)	(0.039)	(0.125)
*Number of physicians per 1000 deliveries*	0.000	0.004*	0.003
p value	(0.769)	(0.045)	(0.239)
95% CI	[− 0.001, 0.001]	[0.000, 0.007]	[− 0.002, 0.007]
SE	(0.001)	(0.002)	(0.002)
*Number of midwives per 1000 deliveries*	0.000	0.002	0.003
p value	(0.884)	(0.336)	(0.150)
95% CI	[− 0.001, 0.001]	[− 0.002, 0.006]	[− 0.001, 0.006]
SE	(0.001)	(0.002)	(0.002)
*Number of deliveries per 1000*	− 0.006*	− 0.013	− 0.008
p value	(0.037)	(0.670)	(0.844)
95% CI	[− 0.012, 0.000]	[− 0.074, 0.048]	[− 0.089, 0.072]
SE	(0.003)	(0.031)	(0.041)
Num. obs	1479	1479	1024
R^2^	0.753	0.012	
R^2^ adj	0.752	− 0.427	
AIC	− 2916.6	− 4015.8	
BIC	− 2884.8	− 3989.3	
Std. errors	HC1	HC1	

^+^p < 0.1, *p < 0.05, **p < 0.01, ***p < 0.001

**Table 14:** Uni- and multivariate panel models with two-ways difference generalized method of moment estimators on risk-adjusted C-section ratio

	Ratio of observed to expected (O/E) cesarean births (2015–2019) (two-way fixed effects model)		
	Univariate model	Multivariate model	Multivariate model with control variables
*Lagged ratio of observed to expected ratio (O/E) of cesarean births*	0.544***	0.534***	0.537***
p value	(< 0.001)	(< 0.001)	(< 0.001)
95% CI	[0.299, 0.790]	[0.293, 0.776]	[0.293, 0.781]
SE	(0.130)	(0.128)	(0.130)
*Number of physicians per 1000 deliveries*	0.004	0.002	0.002
p value	(0.107)	(0.293)	(0.313)
95% CI	[− 0.001, 0.008]	[− 0.002, 0.007]	[− 0.002, 0.007]
SE	(0.002)	(0.002)	(0.002)
*Number of midwives per 1000 deliveries*		0.003	0.003
p value		(0.192)	(0.182)
95% CI		[− 0.001, 0.006]	[− 0.001, 0.006]
SE		(0.002)	(0.002)
*Number of deliveries per 1000*		− 0.013	− 0.012
p value		(0.759)	(0.777)
95% CI		[− 0.094, 0.068]	[− 0.093, 0.069]
SE		(0.042)	(0.042)
*Ownership: public (ref. category: ownership private)*			− 0.047
p value			(0.356)
95% CI			[− 0.142, 0.049]
SE			(0.050)
*Ownership: non-profit (ref. category: ownership private)*			− 0.025
p value			(0.655)
95% CI			[− 0.130, 0.081]
SE			(0.055)
*Perinatal centers level I (care level 1) (ref. category: regular obstetric department (care level 4))*			0.054
p value			(0.150)
95% CI			[− 0.020, 0.128]
SE			(0.038)
*Perinatal centers level II (care level 2) (ref. category: regular obstetric department (care level 4)*			0.023
p value			(0.648)
95% CI			[− 0.074, 0.121]
SE			(0.051)
*Perinatal focus (care level 3) (ref. category: regular obstetric department (care level 4)*			− 0.030
p value			(0.342)
95% CI			[− 0.091, 0.032]
SE			(0.031)
*Teaching status: academic teaching hospital (ref. category: teaching status: no teaching assignment)*			− 0.004
p value			(0.863)
95% CI			[− 0.054, 0.046]
SE			(0.025)
*Teaching status: University Hospital (ref. category: teaching status: no teaching assignment)*			− 0.003
p value			(0.918)
95% CI			[− 0.056, 0.051]
SE			(0.027)
Num. obs	1031	1031	1031
Hansen–Sargan test/J test (p value)	3.351 (0.646)	3.415 (0.636)	3.710 (0.592)
Arellano–Bond test/autocorrelation test (1) (p value)	− 5.461 (< 0.001)	− 5.504 (< 0.001)	− 5.476 (< 0.001)
Arellano–Bond test/autocorrelation test (2) (p value)	− 0.088 (0.930)	− 0.086 (0.931)	− 108 (0.914)
Wald test for coefficients (p value)	21.518 (< 0.001)	23.198 (< 0.001)	53.958 (< 0.001)
Wald test for time dummies (p value)	2.385 (0.497)	2.119 (0.379)	1.982 (0.576)
Std. errors	HC1	HC1	HC1

^+^p < 0.1, *p < 0.05, **p < 0.01, ***p < 0.001

**Table 15:** Uni- and multivariate panel models with two-way fixed effects on risk-adjusted c-section ratio in solo obstetric departments

	Ratio of observed to expected (O/E) cesarean births (2015–2019) (two-way fixed effects model) in solo obstetric departments			
	Univariate models	Univariate models	Univariate models	Multivariate models
*Number of physicians per 1000 deliveries*	− 0.003			− 0.003
p value	(0.562)			(0.546)
95% CI	[− 0.013, 0.007]			[− 0.013, 0.007]
SE	(0.005)			(0.005)
*Number of midwives per 1000 deliveries*		0.001		0.001
p value		(0.699)		(0.710)
95% CI		[− 0.006, 0.009]		[− 0.006, 0.009]
SE		(0.004)		(0.004)
*Number of deliveries per 1000*			− 0.009	− 0.008
p value			(0.865)	(0.875)
95% CI			[− 0.107, 0.090]	[− 0.111, 0.095]
SE			(0.050)	(0.052)
Num. obs	308	308	308	308
R^2^	0.003	0.002	0.000	0.005
R^2^ adj	− 0.539	− 0.539	− 0.542	− 0.550
AIC	− 967.4	− 967.2	− 966.7	− 964.2
BIC	− 959.9	− 959.8	− 959.2	− 949.3
Std. errors	HC1	HC1	HC1	HC1

^+^p < 0.1, *p < 0.05, **p < 0.01, ***p < 0.001

**Table 16:** Uni- and multivariate panel models with two-ways random effects on risk-adjusted C-section ratio in solo obstetric departments

	Ratio of observed to expected (O/E) cesarean births (2015–2019) (two-way random effects model) in solo obstetric departments			
	Univariate models			Multivariate model
*Intercept*	0.948***	0.950***	0.965***	0.950***
p value	(< 0.001)	(< 0.001)	(< 0.001)	(< 0.001)
95% CI	[0.862, 1.033]	[0.841, 1.059]	[0.879, 1.052]	[0.737, 1.163]
SE	(0.044)	(0.055)	(0.044)	(0.108)
*Number of physicians per 1000 deliveries*	0.001			0.001
p value	(0.837)			(0.874)
95% CI	[− 0.008, 0.010]			[− 0.010, 0.012]
SE	(0.005)			(0.005)
*Number of midwives per 1000 deliveries*		0.000		0.000
p value		(0.927)		(0.946)
95% CI		[− 0.010, 0.011]		[− 0.011, 0.012]
SE		(0.005)		(0.006)
*Number of deliveries per 1000*			− 0.007	− 0.003
p value			(0.802)	(0.919)
95% CI			[− 0.060, 0.046]	[− 0.067, 0.060]
SE			(0.027)	(0.032)
Num. obs	308	308	308	308
R^2^	0.015	0.004	0.001	0.001
R^2^ adj	0.012	0.001	− 0.002	− 0.009
AIC	− 177.5	− 175.7	− 175.5	− 172.6
BIC	− 166.3	− 164.6	− 164.3	− 154.0
Std. errors	HC1	HC1	HC1	HC1

^+^p < 0.1, *p < 0.05, **p < 0.01, ***p < 0.001

**Table 17:** Uni- and multivariate panel models with two-way fixed effects on crude C-section/birth ratio

	Ratio of C-section to all births (2015–2019) (two-way fixed effects model)			
	Univariate models	Univariate models	Univariate models	Multivariate models
*Number of physicians per 1000 deliveries*	0.002***			0.001*
p value	(< 0.001)			(0.017)
95% CI	[0.001, 0.002]			[0.000, 0.002]
SE	(0.000)			(0.001)
*Number of midwives per 1000 deliveries*		0.001		0.000
p value		(0.148)		(0.571)
95% CI		[0.000, 0.001]		[− 0.001, 0.001]
SE		(0.000)		(0.000)
*Number of deliveries per 1000*			− 0.024**	− 0.013
p value			(0.001)	(0.118)
95% CI			[− 0.039, − 0.009]	[− 0.029, 0.003]
SE			(0.007)	(0.008)
Num. obs	2089	2089	2089	2089
R^2^	0.011	0.003	0.008	0.013
R^2^ adj	− 0.320	− 0.330	− 0.324	− 0.318
AIC	− 10,093.2	− 10,076.2	− 10,086.1	− 10,094.7
BIC	− 10,081.9	− 10,064.9	− 10,074.8	− 10,072.1
Std. errors	HC1	HC1	HC1	HC1

^+^p < 0.1, *p < 0.05, **p < 0.01, ***p < 0.001

**Table 18:** Endogeneity check for instrument variable on crude C-section/birth ratio

	Ratio of C-section to all births (2015–2019) (two-way fixed effects model)	
	univariate model	multivariate model
*Number of physicians per 1000 deliveries*	0.004***	0.006***
p value	(< 0.001)	(< 0.001)
95% CI	[0.002, 0.006]	[0.004, 0.008]
SE	(0.001)	(0.001)
*Number of midwives per 1000 deliveries*		0.000
p value		(0.443)
95% CI		[− 0.001, 0.001]
SE		(0.001)
*Number of deliveries per 1000*		0.031***
p value		(< 0.001)
95% CI		[0.022, 0.040]
SE		(0.005)
*Endogenous part from the IV*	− 0.003*	− 0.003**
p value	(0.015)	(0.008)
95% CI	[− 0.005, 0.000]	[− 0.005, − 0.001]
SE	(0.001)	(0.001)
Num. obs	2037	2037
R^2^	0.032	0.102
R^2^ adj	− 0.291	− 0.199
AIC	− 7520.7	− 7669.4
BIC	− 7503.8	− 7641.3
Std. errors	HC1	HC1

^+^p < 0.1, *p < 0.05, **p < 0.01, ***p < 0.001

**Table 19:** Dynamic IV models with system generalized method of moment estimators on crude C-section/birth ratio

	Ratio of observed to expected (O/E) cesarean births (2015–2019) (two-way fixed effects model)		
	Univariate model	Multivariate model	Multivariate model with control variables
*Lagged C-section to delivery ratio*	0.426***	0.518***	0.530***
p value	(< 0.001)	(< 0.001)	(< 0.001)
95% CI	[0.273, 0.578]	[0.392, 0.644]	[0.416, 0.643]
SE	(0.075)	(0.062)	(0.061)
*Number of physicians per 1000 deliveries*	0.001**	0.002***	0.001**
p value	(0.002)	(< 0.001)	(0.007)
95% CI	[0.000, 0.002]	[0.001, 0.003]	[0.000, 0.002]
SE	(0.000)	(0.000)	(0.000)
*Number of midwives per 1000 deliveries*		0.000	0.000
p value		(0.689)	(0.396)
95% CI		[− 0.001, 0.001]	[− 0.001, 0.000]
SE		(0.000)	(0.000)
*Number of deliveries per 1000*		0.009**	− 0.010**
p value		(0.002)	(0.006)
95% CI		[0.003, 0.014]	[− 0.016, − 0.004]
SE		(0.003)	(0.004)
*Ownership: public (ref. category: ownership private)*			− 0.004
p value			(0.434)
95% CI			[− 0.013, 0.005]
SE			(0.005)
*Ownership: non-profit (ref. category: ownership private)*			0.001
p value			(0.835)
95% CI			[− 0.008, 0.010]
SE			(0.005)
*Perinatal centers level I (care level 1) (ref. category: regular obstetric department (care level 4))*			0.035***
p value			(< 0.001)
95% CI			[0.022, 0.048]
SE			(0.007)
*Perinatal centers level II (care level 2) (ref. category: regular obstetric department (care level 4)*			0.021***
p value			(< 0.001)
95% CI			[0.010, 0.032]
SE			(0.006)
*Perinatal focus (care level 3) (ref. category: regular obstetric department (care level 4)*			0.002
p value			(0.570)
95% CI			[− 0.005, 0.010]
SE			(0.004)
*Teaching status: academic teaching hospital (ref. category: Teaching status: no teaching assignment)*			0.001
p value			(0.711)
95% CI			[− 0.006, 0.009]
SE			(0.004)
*Teaching status: University Hospital (ref. category: teaching status: no teaching assignment)*			0.012
p value			(0.154)
95% CI			[− 0.002, 0.026]
SE			(0.009)
Num. obs	2520	2520	2520
Hansen–Sargan test/J test (p value)	10.672 (0.299)	21.704 (0.027)	21.474 (0.256)
Arellano–Bond test/autocorrelation test (1) (p value)	− 5.698 (< 0.001)	− 6.056 (< 0.001)	− 6.031 (< 0.001)
Arellano–Bond test/autocorrelation test (2) (p value)	− 0.607 (0.544)	− 0.284 (0.777)	− 0.400 (0.689)
Wald test for coefficients (p value)	53.519 (< 0.001)	136.851 (< 0.001)	381.974 (< 0.001)
Wald test for time dummies (p value)	7.748 (0.052)	5.141 (0.162)	8.580 (0.035)
Std. errors	HC1	HC1	HC1

^+^p < 0.1, *p < 0.05, **p < 0.01, ***p < 0.001

**Table 20:** Comparison of effect size lagged variable for OLS, fixed effect and difference GMM estimator on crude C-section/birth ratio

	Ratio of C-section to all births (2015–2019) (two-way fixed effects model)		
	OLS	Panel model with fixed effects	Difference GMM
*Intercept*	0.029***		
p value	(< 0.001)		
95% CI	[0.021, 0.037]		
SE	(0.004)		
*Lagged C-section to delivery ratio*	0.876***	− 0.007	0.503***
p value	(< 0.001)	(0.826)	(< 0.001)
95% CI	[0.854, 0.898]	[− 0.074, 0.059]	[0.289, 0.716]
SE	(0.011)	(0.034)	(0.109)
*Number of physicians per 1000 deliveries*	0.000	0.001^+^	0.001
p value	(0.167)	(0.055)	(0.413)
95% CI	[0.000, 0.001]	[0.000, 0.002]	[− 0.001, 0.002]
SE	(0.000)	(0.001)	(0.001)
*Number of midwives per 1000 deliveries*	0.000	0.001	0.001
p value	(0.421)	(0.160)	(0.336)
95% CI	[0.000, 0.001]	[0.000, 0.002]	[− 0.001, 0.002]
SE	(0.000)	(0.001)	(0.001)
*Number of deliveries per 1000*	0.001	− 0.014	− 0.014
p value	(0.183)	(0.201)	(0.447)
95% CI	[− 0.001, 0.004]	[− 0.036, 0.008]	[− 0.050, 0.022]
SE	(0.001)	(0.011)	(0.018)
Num. obs	1489	1489	
R^2^	0.799	0.020	
R^2^ adj	0.798	− 0.416	
AIC	− 6218.6	− 7555.6	
BIC	− 6186.8	− 7529.0	
Std. errors	HC1	HC1	

^+^p < 0.1, * p < 0.05, ** p < 0.01, *** p < 0.001

**Table 21:** Uni- and multivariate panel models with two-ways difference generalized method of moment estimators on crude C-section/birth ratio

	Ratio of C-section to all births (2015–2019) (two-way fixed effects model)		
	Univariate model	Multivariate model	Multivariate model with control variables
*Lagged C-section to delivery ratio*	0.522***	0.503***	0.511***
p value	(< 0.001)	(< 0.001)	(< 0.001)
95% CI	[0.310, 0.734]	[0.289, 0.716]	[0.295, 0.727]
SE	(0.108)	(0.109)	(0.110)
*Number of physicians per 1000 deliveries*	0.001	0.001	0.001
p value	(0.117)	(0.413)	(0.433)
95% CI	[0.000, 0.003]	[− 0.001, 0.002]	[− 0.001, 0.002]
SE	(0.001)	(0.001)	(0.001)
*Number of midwives per 1000 deliveries*		0.001	0.001
p value		(0.336)	(0.310)
95% CI		[− 0.001, 0.002]	[− 0.001, 0.002]
SE		(0.001)	(0.001)
*Number of deliveries per 1000*		− 0.014	− 0.013
p value		(0.447)	(0.471)
95% CI		[− 0.050, 0.022]	[− 0.050, 0.023]
SE		(0.018)	(0.019)
*Ownership: public (ref. category: ownership private)*			0.000
p value			(0.995)
95% CI			[− 0.051, 0.051]
SE			(0.026)
Ownership: non-profit (ref. category: ownership private)			0.003
p value			(0.899)
95% CI			[− 0.038, 0.043]
SE			(0.021)
*Perinatal centers level I (care level 1) (ref. category: regular obstetric department (care level 4))*			0.009
p value			(0.367)
95% CI			[− 0.011, 0.029]
SE			(0.010)
*Perinatal centers level II (care level 2) (ref. category: regular obstetric department (care level 4)*			0.008
p value			(0.594)
95% CI			[− 0.020, 0.035]
SE			(0.014)
*Perinatal focus (care level 3) (ref. category: regular obstetric department (care level 4)*			− 0.011
p value			(0.334)
95% CI			[− 0.032, 0.011]
SE			(0.011)
*Teaching status: academic teaching hospital (ref. category: teaching status: no teaching assignment)*			− 0.006
p value			(0.509)
95% CI			[− 0.022, 0.011]
SE			(0.008)
Teaching status: University Hospital (ref. category: teaching status: no teaching assignment)			− 0.004
p value			(0.675)
95% CI			[− 0.021, 0.013]
SE			(0.009)
Num. obs	1031	1031	1031
Hansen–Sargan test/J test (p value)	1.937 (0.858)	1.704 (0.888)	1.685 (0.891)
Arellano–Bond test/autocorrelation test (1) (p value)	− 6.098 (< 0.001)	− 6.225 (< 0.001)	− 6.240 (< 0.001)
Arellano–Bond test/autocorrelation test (2) (p value)	− 0.365 (0.715)	− 0.494 (0.622)	− 0.405 (0.686)
Wald test for coefficients (p value)	28.950 (< 0.001)	30.083 (< 0.001)	56.313 (< 0.001)
Wald test for time dummies (p value)	9.975 (0.019)	9.845 (0.020)	9.890 (0.020)
Std. errors	HC1	HC1	HC1

^+^p < 0.1, *p < 0.05, **p < 0.01, ***p < 0.001

**Table 22:** Example of a depiction for calculating the expected risk-adjusted cesarean section rate by IQTIG for 2018. Own translation. (Source: https://iqtig.org/downloads/auswertung/auswertung/2018/16n1gebh/QSKH_16n1-GEBH_2018_QIDB_V02_2019-04-11.pdf, p. 19)

Reference probability: 14.205% (odds: 0.165)					
Risk factor	Regression coefficient	Standard error	Z value	Odds ratio	95% confidence interval
Constant	− 1.798342673800550	0.004	− 408.407	–	–
Age 35–38 years	0.034231814566637	0.008	4.439	1.035	1.019–1.051
Age > 38	0.286484745408656	0.011	24.971	1.332	1.302–1.362
Birth risk: amnion infection syndrome (suspected)	2.647566531445380	0.040	66.591	14.120	13.061–15.264
Birth risk: diabetes mellitus	0.354402849335064	0.014	24.645	1.425	1.386–1.466
Birth risk: premature birth	0.356693940042668	0.018	20.341	1.429	1.380–1.479
Birth risk: hypertensive Pregnancy disorder or HELLP Syndrome	1.471801217071380	0.018	81.161	4.357	4.205–4.515
Birth risk: pathological CTG. poor fetal heart sounds. or acidosis during birth (detected by FBS)	0.922892533667843	0.007	124.693	2.517	2.480–2.553
Birth risk: placenta praevia	3.414601549901360	0.061	55.693	30.405	26.962–34.287
Birth risk: breech position	3.585447507902870	0.018	199.288	36.069	34.820–37.364
Birth risk: face/forehead Presentation	1.942056761249630	0.063	30.713	6.973	6.160–7.893
Birth risk: transverse/oblique position	6.515521815847840	0.268	24.293	675.546	399.356–1142.746
Birth risk: previous cesarean section or other uterus operations	1.994488346490800	0.016	122.135	7.348	7.117–7.587
Multiple pregnancy	1.453148908289270	0.024	59.963	4.277	4.078–4.485
Mother's record: hypertension or proteinuria	0.241446328929522	0.025	9.548	1.273	1.212–1.338
Mother's record: placental insufficiency	0.735298957679869	0.031	23.853	2.086	1.964–2.216
Mother's record: previous cesarean section or uterus operations	0.294514404293640	0.016	17.888	1.342	1.300–1.387

## Data Availability

The analysis provided relied upon the hospital quality reports from German acute care hospitals. All the data utilized in this analysis is accessible to the public and can be found at www.g-ba.de/qualitaetsberichte.
